# Structural and Resting State Functional Connectivity of the Subthalamic Nucleus: Identification of Motor STN Parts and the Hyperdirect Pathway

**DOI:** 10.1371/journal.pone.0039061

**Published:** 2012-06-29

**Authors:** Ellen J. L. Brunenberg, Pim Moeskops, Walter H. Backes, Claudio Pollo, Leila Cammoun, Anna Vilanova, Marcus L. F. Janssen, Veerle E. R. M. Visser-Vandewalle, Bart M. ter Haar Romeny, Jean-Philippe Thiran, Bram Platel

**Affiliations:** 1 Department of Biomedical Engineering, Eindhoven University of Technology, Eindhoven, The Netherlands; 2 Department of Radiology, Maastricht University Medical Center, Maastricht, The Netherlands; 3 School for Mental Health and NeuroScience, Maastricht University, Maastricht, The Netherlands; 4 Signal Processing Institute, École Polytechnique Fédérale de Lausanne, Lausanne, Switzerland; 5 Department of Neurosurgery, Lausanne University Hospital (CHUV), Lausanne, Switzerland; 6 Department of Neurosurgery, Maastricht University Medical Center, Maastricht, The Netherlands; 7 MIND, Maastricht University Medical Center, Maastricht, The Netherlands; 8 Fraunhofer MEVIS, Bremen, Germany; Institute of Automation, Chinese Academy of Sciences, China

## Abstract

Deep brain stimulation (DBS) for Parkinson’s disease often alleviates the motor symptoms, but causes cognitive and emotional side effects in a substantial number of cases. Identification of the motor part of the subthalamic nucleus (STN) as part of the presurgical workup could minimize these adverse effects. In this study, we assessed the STN’s connectivity to motor, associative, and limbic brain areas, based on structural and functional connectivity analysis of volunteer data. For the structural connectivity, we used streamline counts derived from HARDI fiber tracking. The resulting tracks supported the existence of the so-called “hyperdirect” pathway in humans. Furthermore, we determined the connectivity of each STN voxel with the motor cortical areas. Functional connectivity was calculated based on functional MRI, as the correlation of the signal within a given brain voxel with the signal in the STN. Also, the signal per STN voxel was explained in terms of the correlation with motor or limbic brain seed ROI areas. Both right and left STN ROIs appeared to be structurally and functionally connected to brain areas that are part of the motor, associative, and limbic circuit. Furthermore, this study enabled us to assess the level of segregation of the STN motor part, which is relevant for the planning of STN DBS procedures.

## Introduction

### Background

Deep brain stimulation (DBS) of the subthalamic nucleus (STN) is an important therapy for Parkinson’s disease (PD) [1], offering significant and sustained improvement of motor symptoms [2]–[4]. However, stimulation-induced cognitive alterations and psychiatric side effects occur in a substantial number of cases [5]–[10]. Current spread to the associative and limbic pathways running through the STN explains these side effects [11], though dopaminergic withdrawal and premorbid neuropsychiatric vulnerability play a role as well. Accurate targeting and selective stimulation of the STN motor area seem essential, both to achieve the optimal effect on the motor symptoms [12], [13] and to minimize the adverse effects.

Based on topical literature, the STN is divided into three functionally different parts, distinguished by their afferent and efferent connections in the non-human primate [14]. The largest part is the sensorimotor area, which encompasses the dorsolateral two-thirds of the STN. The associative area is located in the ventrolateral STN, while the smallest part, namely the limbic area, is positioned at the medial tip of the STN [11], [14], [15]. Although the literature presents the motor, associative, and limbic cortico-basal-ganglia loops as parallel circuits, it is still not obvious to what extent these functional circuits are integrated within the STN. The possibility of selective stimulation of the motor STN, without affecting the associative and limbic circuits, is strongly influenced by the level of integration of these loops within the STN.

To resolve these issues, we looked into MRI methods providing functional information for the identification of the STN parts. In the study described in this article, we investigated the structural connectivity of the STN based on diffusion-weighted MRI. In addition, resting state BOLD functional MRI (fMRI) enabled us to examine the functional connectivity. The results provide us with more insight on the level of segregation of the motor and non-motor cortico-basal-ganglia loops at the level of the STN.

### Related Work

Structural connectivity analysis of brain networks based on diffusion-weighted MRI has been performed for about 8 years [16]–[21]. In their review [22], Behrens and Johansen-Berg discussed three methods for parcellation of gray matter nuclei. First, parcellation can be done without any prior knowledge about projections, using changes in connectivity profiles per voxel [23]. Second, local diffusion profiles can be clustered to separate regions [24]–[26]. Third and most common, prior knowledge about projections (from atlases or fMRI) can be used. This method has been practiced for the thalamus [27], striatum [28]–[30], and the combination of thalamus, striatum and globus pallidus [31], [32].

To our knowledge, no studies have been published that analyze the full structural connectivity of the STN. Aron et al. analyzed solely the pathways between the STN and the inferior frontal cortex and pre-supplementary motor area [33], while Forstmann et al. considered only the connectivity of the STN with the pre-supplementary motor area, primary motor cortex, anterior cingulate cortex, inferior frontal gyrus, and the striatum [34].

With respect to functional connectivity, a number of studies have already applied fMRI-based functional connectivity analysis to the basal ganglia. Some investigated the motor network in healthy subjects [35] or patients with PD [36], without looking at the STN specifically. Others examined the functional connectivity of specific nuclei such as the red nucleus [37] or the striatum [38]. Barnes et al. [39] identified subdivisions in the caudate and putamen based on functional connectivity data.

As far as we know, the only resting state functional connectivity study concentrating on the STN was reported by Baudrexel et al. [40], [41]. However, they reported only on alterations in the functional connectivity pattern caused by PD and did not discuss the ‘normative’ functional connectivity of the STN. Other studies concerning STN connectivity used more invasive techniques, such as PET [42]–[44] and electrophysiological recordings in humans [44]–[46] and in the mouse brain [47].

### Aim

As a complement to the mentioned literature, a complete description of the structural and functional connectivity of the STN, based on non-invasive data, is useful. In addition, segmentation of the STN motor part based on connectivity analysis has not been attempted before. The aim of this study was to assess the STN’s structural and functional connectivity in healthy subjects based on fiber tracking derived from high angular resolution diffusion imaging (HARDI) data and correlation analysis using BOLD fMRI data, respectively.

We hypothesized that the results would offer insight into the level of segregation of the STN motor area and the feasibility of selective stimulation of this part. In addition, we assumed that the results would provide evidence for the existence of the “hyperdirect” pathway in humans.

## Methods

### Data Acquisition and Preprocessing

#### Ethics statement

A group of 12 healthy adult subjects (5 males, 7 females, age 24–49 years, mean age = 29.9) was recruited from Eindhoven University of Technology and Maastricht University Medical Center. Written informed consent was obtained from all subjects, and the study was approved by the Medical Ethics Committee of Maastricht University Medical Center.

#### Data acquisition

Data acquisition was done on a Philips Achieva 3 T system. Structural images were scanned using a three-dimensional inversion recovery (IR) 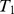-weighted sequence (including 60 coronal slices) and a three-dimensional turbo spin-echo (TSE) 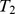-weighted sequence (50 coronal slices). HARDI scanning was performed using a diffusion-weighted EPI protocol, acquiring a series of 128 diffusion-weighted images with different gradient directions and 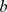-value 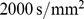, together with an unweighted 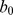 image. Functional imaging was done using a blood-oxygen-level-dependent (BOLD) contrast sensitive EPI protocol, acquiring one dynamic run of 200 time points. The TSE and fMRI measurements both covered only part of the brain (coronal FOV of 50 mm for TSE and 75 mm for fMRI), situated around the midbrain region of interest, parallel to the brain stem. Detailed scanning parameters can be found in Table 1.

**Table 1:** MRI parameters.

Parameter	Inversion recovery	Turbo spin-echo	Diffusion-weighted	Functional
TE (ms)	15	110	85	35
TR (ms)	5441	2500	6370	2200
Direction	coronal	coronal	axial	coronal
Number of slices	60	50	52	25
Slice thickness (mm)	3.0	1.0	2.0	3.0
Number of voxels	640×640	256×256	128×128	128×128
Voxel size (mm)	0.359×0.359	1.0×1.0	2.0×2.0	1.563×1.563
Scan duration	7 min 26 s	7 min 43 s	14 min 39 s	7 min 20 s

Parameters of MRI sequences used for this study.

The first four volunteers underwent HARDI scans with more basic parameters than described above, which is why structural data analysis was performed using the data of the remaining 8 healthy adult subjects (4 males, 4 females, age 24–49 years, mean age = 31.5). The functional scans of two volunteers displayed too many motion artifacts, thus functional data analysis was executed on a group of 10 subjects (4 males, 6 females, age 24–35 years, mean age = 27.5).

#### Data preprocessing

**Figure 1:**
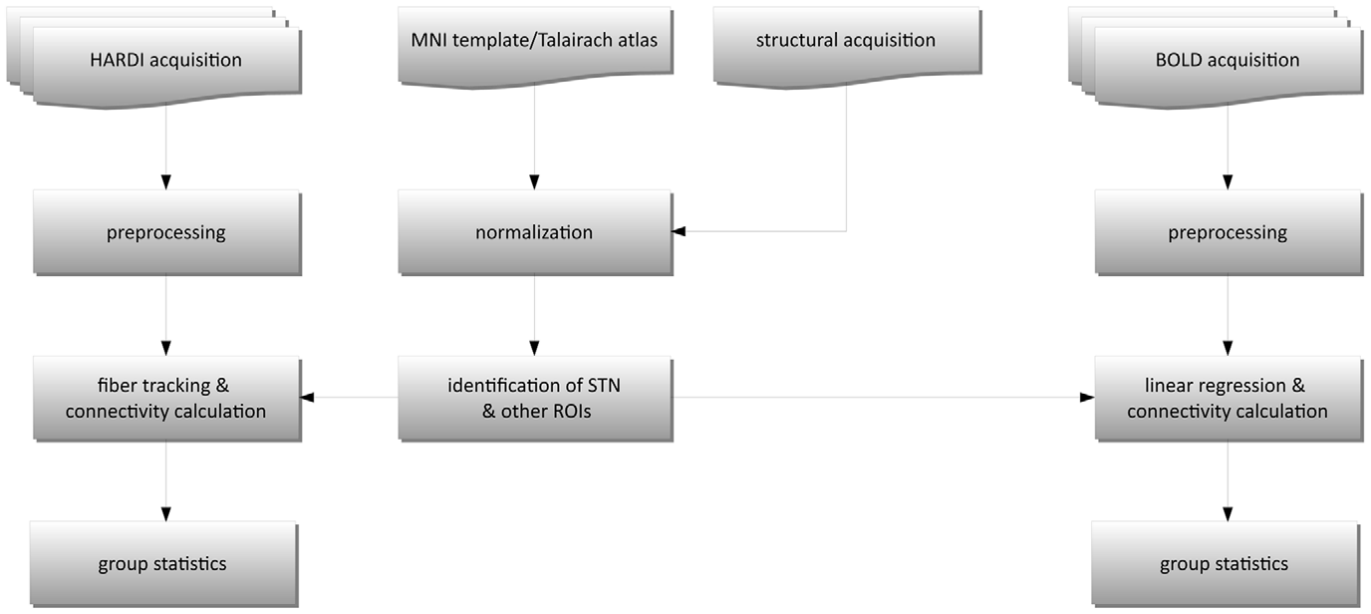
Flowchart of data analysis steps for structural and functional connectivity.

A flowchart representing the used data analysis pipeline is shown in Figure 1. After acquisition, the data were preprocessed to reduce artifacts. All HARDI images were registered to the 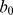 image using FSL’s [48] eddy current correction, in order to correct for distortions and head motion. Subsequently, Q-ball estimation [49] was used to prepare the data for fiber tracking (this will be explained further on). Preprocessing of the BOLD images was also meant to reduce signal variance due to factors other than neuronal activation. This included (i) correction for head movement using MCFLIRT [50] and brain extraction with BET [51] in FSL, (ii) removal of the first 5 time points to correct for 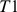-saturation effects, (iii) slice timing correction and spatial smoothing (3 mm FWHM) (both in SPM5), (iv) linear detrending and temporal bandpass filtering (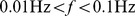) (both using the REST toolbox [52]).

All data were registered to match the MNI152 template [53] and the accompanying Talairach atlas [54], [55], as provided by FSL. For the HARDI pipeline, the MNI152 template was affinely registered to the 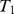-weighted IR images by FSL’s FLIRT [56] (9 DOF; mutual information). Subsequently, a nonlinear transformation using FNIRT [57] was applied. In addition, intersubject affine registration of the IR data to the unweighted (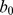) image was done in the same way as described above. Finally, all transformations were sequentially applied to the MNI152 template and the accompanying atlas labels, interpolating the latter in a nearest neighbor fashion. We chose to apply the transformations to the atlas images to avoid deformation of the HARDI data. As for the BOLD images, the anatomical images were rigidly registered to the functional data using FLIRT [56] (6 DOF). The data from different subjects were spatially normalized by means of affine registration with the MNI152 template and the atlas labels, also in FLIRT. In each subject, the subthalamic nucleus ROIs were defined by the voxels with the right and left STN labels in the registered Talairach atlas.

### Structural Connectivity Analysis

#### Probabilistic tractography

To assess the structural connectivity between regions, fiber tracking was performed to estimate the trajectories of the white matter axonal bundles. We employed Camino’s [58] probabilistic tractography, a refined version of the streamline-based probabilistic index of connectivity (PICo) algorithm [59]–[61].

As a preprocessing step, the diffusion profile in each voxel is reconstructed from the HARDI data. A threshold on the 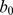 image ensures that profiles are only generated within the brain. Subsequently, the directions of principal diffusion are detected as the peaks in the calculated orientation distribution functions (ODFs). The shape of the ODF peaks is used to estimate a probability distribution function that describes the uncertainty of the principal diffusion directions. The actual tracking procedure starts multiple streamlines from the center of each seed voxel. Each of these streamlines can follow a unique trajectory because the principal diffusion directions are perturbed by the randomly sampled uncertainties.

In this study, we employed Q-ball imaging [49] based on 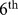 order spherical harmonics for the reconstruction of the orientation distribution functions. As a seed region, we used the STN as labeled in the Talairach atlas and matched to the subject’s HARDI data. Concerning the tracking parameters, we generated 5,000 different streamlines per seed voxel. These were terminated if curvature over a single voxel exceeded 80 degrees, while no threshold was set on anisotropy values and fiber length. The output of the tracking algorithm was saved as raw streamline data in vtk format.

#### Structural connectivity measure

The connectivity between the STN and other gray matter regions in the brain (called target ROIs in the rest of this section) can be calculated based on the fiber tracking output. This output normally consists of as many probability maps as there were seed voxels within the STN. Each of these probability maps contains for every voxel in the brain, the amount of streamlines passing through or ending in that voxel. This amount is expressed as a ratio of the total amount of streamlines starting from the given seed voxel in the STN. A value of 0.5 would therefore mean that 2,500 of the 5,000 streamlines starting in the STN seed voxel pass through or end in the given brain voxel.

However, in a target ROI that consists of more than one voxel, this could lead to biased connectivity calculation. For example, a target ROI where a streamline ends in a voxel on the boundary would then be seen as “less connected” than a target ROI where the streamline ends in the middle of the region. Therefore, we analyzed the streamline data from the fiber tracking algorithm. We accumulated the 5,000 streamlines per seed voxel for all distinct seed voxels of each subject. To calculate the structural connectivity, we followed each streamline in the total set from beginning to end, meanwhile checking the atlas labels of the voxels traversed by the streamline. We considered all voxels with the same label to belong to one target ROI and ensured that each of these regions was counted only once per streamline.

Thus, for every target ROI, we obtained 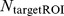, the total number of streamlines passing through or ending in this region. From this number, we calculated our connectivity measure. As streamlines are less likely to reach a target ROI that is far away from the seed region than a nearby ROI, we tried to avoid this bias by taking into account the streamline length:
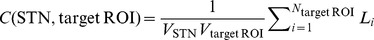
with 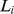 the distance along the 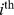 streamline between the STN and the first voxel of the target ROI. The number of streamlines 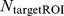 was normalized by 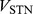 and 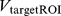. 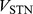 is the size of the STN region derived from the registered atlas (i.e., the number of seed voxels). 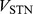 varied between subjects, from 12 to 22 for the left STN (mean size = 16 voxels), and from 13 to 18 for the right STN (mean size = 15 voxels). 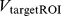 represents the size in voxels of the target region of interest.

**Figure 2:**
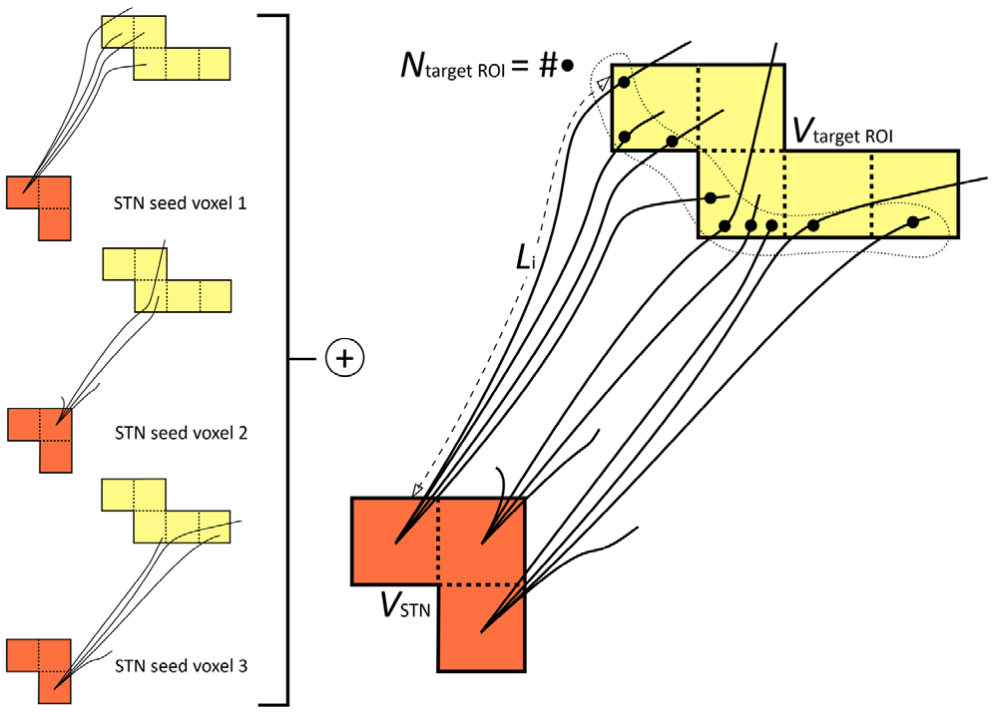
Illustration of the streamline counting per region of interest involved in the calculation of the connectivity measure 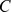.

The calculation of the connectivity measure 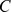 is illustrated in Figure 2. The calculation was done for the target regions of interest of all 8 subjects. To test the statistical significance over this group of subjects, we performed a one-sided Student 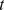-test (test if 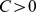).

#### Structural connectivity per voxel of the STN

To examine the possibility to distinguish the STN motor part from the associative and limbic territories, we looked at the motor connectivity for each STN voxel separately. We assessed the connectivity measure 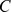 between the STN and four different motor cortical areas: the primary motor cortex (Brodmann area 4), the pre- and supplementary motor areas (Brodmann area 6), the precentral gyrus, and these three regions together. After registration to the MNI152 template (using linear interpolation), the resulting maps were cumulated over all subjects and masked by the atlas STN.

### Functional Connectivity Analysis

#### Whole-brain linear regression

To analyze the STN’s functional connectivity, whole-brain correlation maps were generated by linear regression. Let 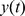 be the resting state signal over time in an arbitrary voxel within the brain. Then 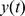 can be expressed as a linear combination of the signal in the STN ROI and some confounds.

The BOLD fMRI signal at time 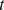, averaged over all voxels of the STN ROI, is denoted by 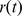. This signal can be standardized according to 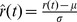, where 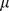 is the mean and 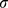 the standard deviation of the signal. So, 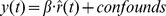, with 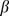 the “goodness of fit” and an estimate of functional connectivity. The confounds include an offset (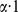), the motion correction parameters from our preprocessing (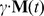), and the global mean signal over all brain voxels (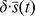). We did not include a regressor for low frequency drift, because we already detrended the data during preprocessing.

Taken together, we performed linear regression using the following system for each STN ROI:
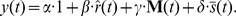



We implemented this linear regression system in the MATLAB programming environment. After performing the regression algorithm, we saved the 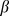 maps for further (statistical) analysis.

#### Statistical analysis

For each voxel within the atlas’ brain mask, not labeled as white matter or cerebrospinal fluid, we performed a Student 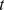-test on the 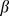 maps for the same STN ROI across subjects. The regression coefficients were normalized first, using the Fisher 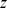-transform. The resulting test statistic 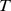 for each voxel was corrected for multiple comparisons using the cluster thresholding method based on random field theory (implemented in the fmristat toolbox for Matlab [62]). A critical cluster size was calculated for test statistics larger than a given threshold, for a given significance level. We used threshold 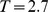 at 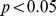, resulting in a critical cluster size of 14 voxels. We separated the thresholding procedure for voxels with negative regression coefficients (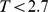) and voxels with positive regression coefficients (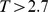). This procedure resulted in significant clusters of voxels with negative and positive regression coefficients, respectively, instead of clusters with mixed responses. The locations of the significant clusters were compared with Talairach atlas labels to generate lists of functionally connected regions.

#### Reverse regression per voxel of the STN

To get more insight in the level of segregation of the STN motor area, and thus to what extent selective stimulation of this part is feasible, we performed a reverse regression procedure. We chose the primary motor cortex, precentral gyrus, and premotor and supplementary motor area as ROIs representing the motor loop, while the hippocampus, amygdala, parahippocampal gyrus, anterior cingulate, and cingulate gyrus formed the ROIs for the limbic group. Linear regression was performed in the same way as described above, using the average signals of both groups of ROIs as principal regressors (the right motor and limbic ROIs for the right STN voxels, and the left ROIs for the left STN voxels, respectively). This procedure yielded two regression coefficient maps, 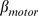 and 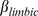 for both the right and left STNs. These maps were registered back towards the MNI152 template (using linear interpolation), masked by the atlas STN ROIs, and summed over all subjects.

## Results

### Structural Connectivity

#### Probabilistic tractography

**Figure 3:**
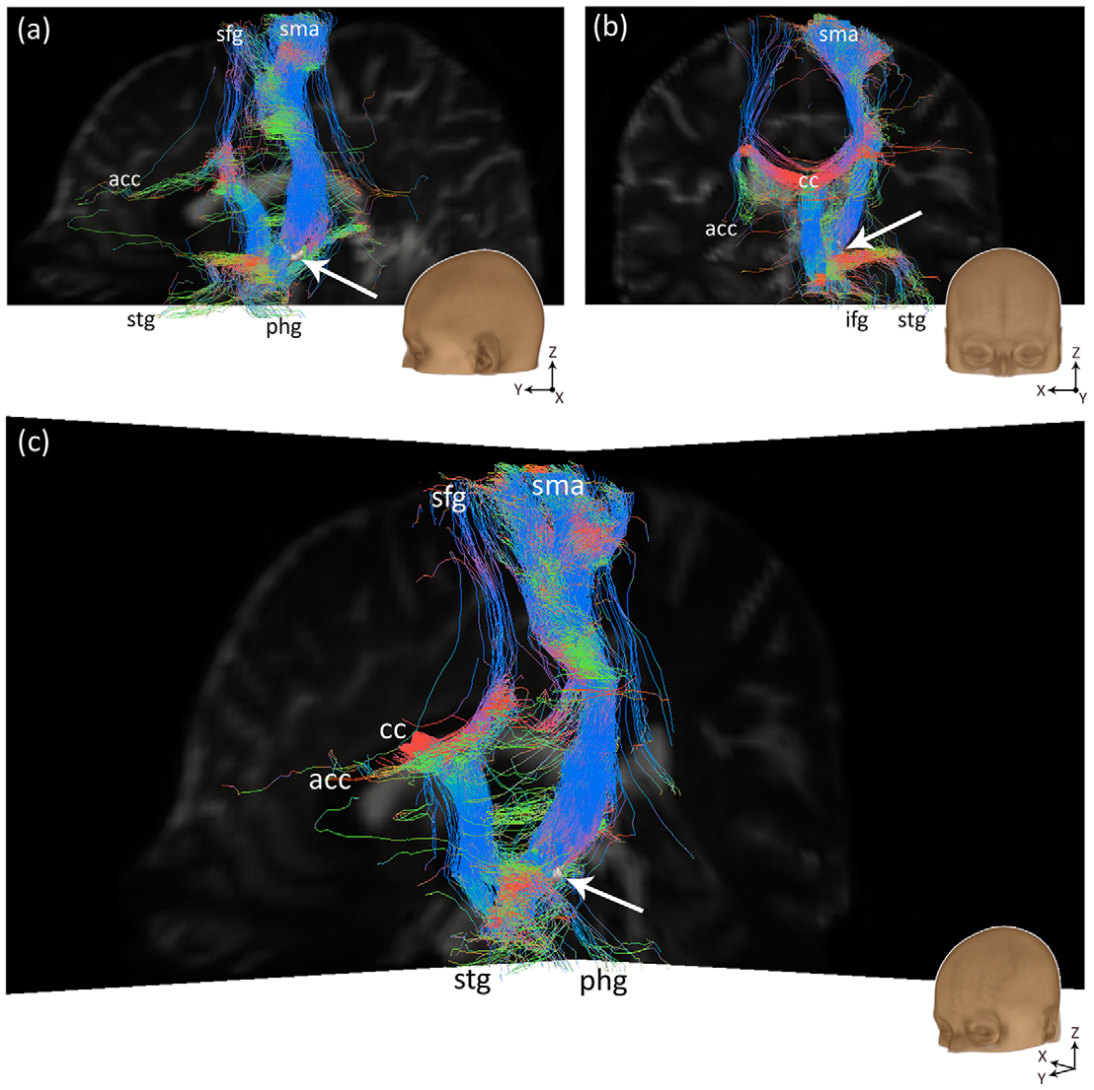
Visualizations of probabilistic fiber tracking results. (a) Sagittal view from the left. (b) Coronal view from the front. (c) Oblique view. The images show 500 streamlines per seed voxel in the right STN of one subject, color-coded for streamline direction (red  =  left-right, green  =  anterior-posterior, blue  =  inferior-superior). The right STN seed is represented by the white surface, indicated by the white arrow. Abbreviations: acc  =  anterior cingulate cortex, cc  =  corpus callosum, ifg  =  inferior frontal gyrus, phg  =  parahippocampal gyrus, sfg  =  superior frontal gyrus, sma  =  supplementary motor area, stg  =  superior temporal gyrus.

After probabilistic tracking, the resulting streamlines were visualized in ParaView [63], an open-source data analysis and visualization application, which allows for interactive data exploration in 3D. For an example, see Figure 3.

**Figure 4:**
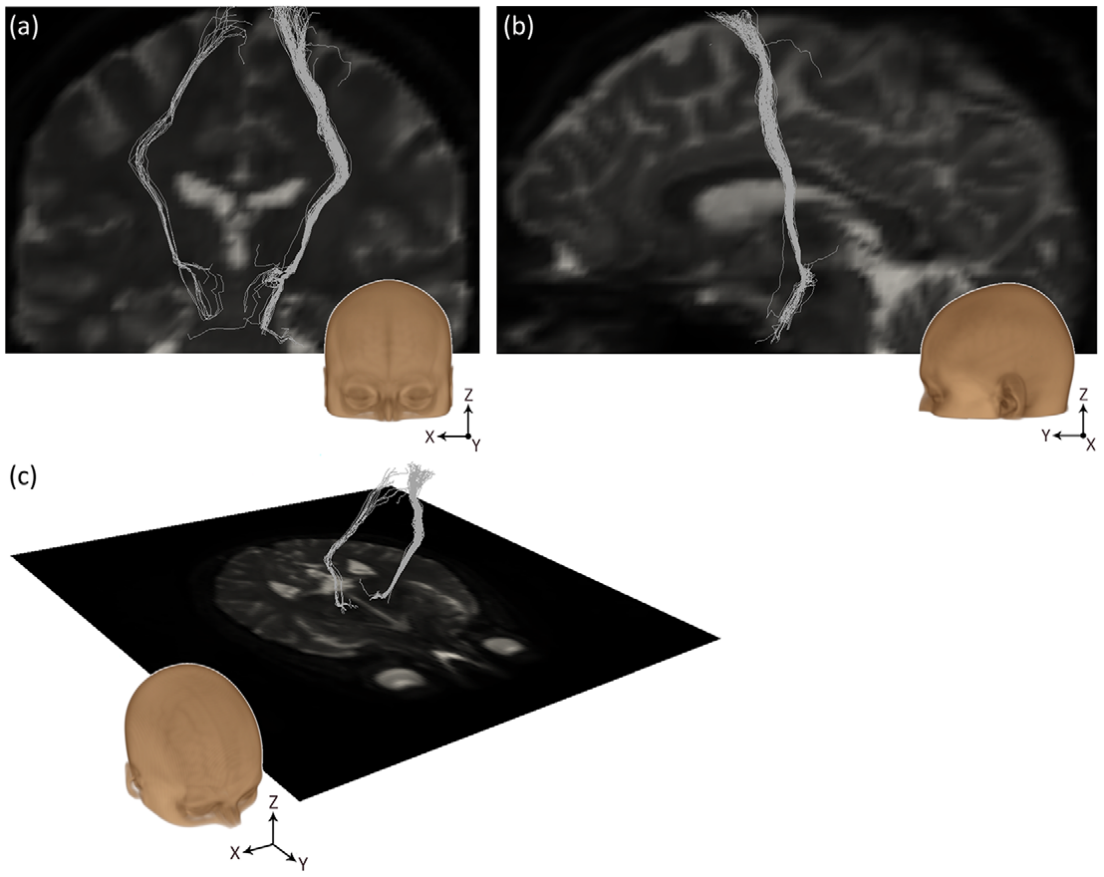
Streamlines from the right and left STN in subject 1, ending in the motor cortex, that do not pass through thalamus, caudate, putamen or globus pallidus. These streamlines are therefore an indication for the existence of the “hyperdirect” pathway. (a) Coronal view. (b) Sagittal view on streamlines from the right STN. (c) Streamline rendering in 3D, showing an axial plane of the unweighted diffusion image.

In addition, we assessed whether the streamline results supported the existence of the so-called “hyperdirect” pathway that directly connects the motor cortex to the STN. For this purpose, we analyzed all streamlines ending in the premotor and supplementary motor cortex, the primary motor cortex and the precentral gyrus and calculated the percentage of the streamlines that did not pass through the thalamus, caudate, putamen or globus pallidus and thus could be said to form a monosynaptic connection between the motor cortex and the STN. Of the 16 analyzed STNs, 10 exhibited direct streamlines to the motor cortical areas. For 3 STNs, the results showed streamlines that seemed to be collaterals of the internal capsule, as expected from literature on primate circuits [14], [15]. These streamlines are shown for one subject in Figure 4. A non-existing medial pathway including the corpus callosum was found in 4 cases, while both the correct lateral and the incorrect medial trajectories were found in 3 cases.

#### Structural connectivity measure

The significant results of the calculated connectivity measure 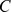, based on streamline tracking in different subjects, are shown in Tables 2 and 3. In the third column of this table, only the most significantly structurally connected regions (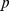 values 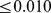) are shown, together with regions that exhibited a significant functional connectivity (their 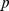 value for structural connectivity was showed as well when available).

**Table 2:** Regions significantly connected to the *right* STN based on structural and functional connectivity measures.

Hemisphere	Region of interest	SC: 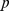 value	FC: 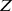 -score(extent)
**SUBCORTICAL**			
Right	Thalamus (Other)	0.000	7.73(171)
Right	Thalamus (Ventral Posterior Lateral)	0.001	
Right	Thalamus (Ventral Anterior)	0.003	
Right	Thalamus (Medial Dorsal)	0.004	
Right	Thalamus (Ventral Posterior Medial)	0.004	
Right	Thalamus (Lateral Posterior)	0.008	
Right	Thalamus (Ventral Lateral)	0.009	
Right	Caudate	0.003	
Right	Putamen	0.004	5.70(22)
Left	Putamen	*0.017*	6.78(86); 5.12(23); 4.81(24)
Right	Lateral Globus Pallidus (GPe)	0.010	
Right	Medial Globus Pallidus (GPi)	0.009	
Right	Red Nucleus	0.003	
Right	Substantia Nigra	0.001	
Right	Claustrum	0.007	
Right	Hypothalamus	0.004	
Right	Midbrain	0.000	−2.73(22)
Left	Midbrain	–	5.90(122)
Right	Pons	–	−2.70(65)
**FRONTAL**			
Right	Precentral Gyrus	–	−2.70(31)
Right	Pre- & Supplementary Motor Area	0.006	
Left	Pre- & Supplementary Motor Area	*0.036*	6.31(85)
Right	Medial Frontal Gyrus	0.010	−2.72(17)
Left	Medial Frontal Gyrus	*0.028*	5.18(19)
Right	Cingulate Gyrus	0.001	
Left	Cingulate Gyrus	*0.045*	3.74(34)
Left	Anterior Cingulate (limbic lobe)	0.010	
Left	Limbic Lobe	0.010	
**PARIETAL**			
Left	Postcentral Gyrus	–	−2.72(22)
**TEMPORAL**			
Right	Superior Temporal Gyrus	0.005	3.95(37); −2.75(19)
Left	Superior Temporal Gyrus	–	7.04(145)
Left	Middle Temporal Gyrus	–	−2.75(15)
Right	Parahippocampal Gyrus	*0.011*	4.13(15); −2.72(35)
Left	Fusiform Gyrus	*0.021*	5.25(16)
**OCCIPITAL**			
Right	Lingual Gyrus (Brodmann 18)	0.008	
Left	Lingual Gyrus	–	4.45(34)
**CEREBELLAR**			
Right	Cerebellum	0.007	3.58(15)
Left	Cerebellum	0.007	5.15(21); 3.74(28)

Regions significantly connected to the *right* STN. The 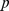-value for the structural connectivity (SC) was calculated using a 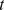-test on 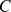, using 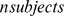 = 8. The 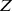-score and cluster extent (in voxels) for the functional connectivity (FC) were determined using correlations with the 10 atlas-based STN ROIs. Here only the regions with 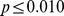 or significant functional connectivity are shown. For the latter cases, the 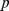-value for structural connectivity was added if lower than 0.050.

**Table 3:** Regions significantly connected to the *left* STN based on structural and functional connectivity measures.

Hemisphere	Region of interest	SC: 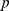 value	FC: 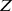 -score(extent)
**SUBCORTICAL**			
Left	Thalamus (Other)	0.000	7.78(39)
Left	Thalamus (Ventral Posterior Lateral)	0.001	
Left	Thalamus (Ventral Anterior)	0.006	
Left	Thalamus (Medial Dorsal)	0.002	
Right	Thalamus (Medial Dorsal)	0.007	
Left	Thalamus (Ventral Posterior Medial)	0.002	
Left	Thalamus (Lateral Posterior)	0.008	
Left	Thalamus (Ventral Lateral)	0.007	
Right	Thalamus (Anterior)	0.005	
Left	Thalamus (Pulvinar)	0.002	
Right	Thalamus (Midline)	0.007	
Left	Thalamus (Mammillary Body)	0.001	
Left	Mammillary Body	0.001	
Right	Caudate	*0.017*	5.71(44)
Left	Putamen	0.007	7.51(76); 4.57(81)
Right	Putamen	–	5.44(81)
Left	Medial Globus Pallidus (GPi)	0.004	
Left	Red Nucleus	0.002	
Right	Red Nucleus	0.005	
Left	Substantia Nigra	0.000	
Left	Hypothalamus	0.008	
Left	Midbrain	0.000	
Right	Midbrain	0.006	4.90(94); −2.71(22)
**FRONTAL**			
Right	Superior Frontal Gyrus	–	−2.71(29); −2.70(23)
Right	Medial Frontal Gyrus	–	3.97(16)
Left	Posterior Cingulate	0.003	
Right	Posterior Cingulate	0.010	
Left	Posterior Cingulate (Brodmann 29)	0.002	
Right	Subcallosal Gyrus (Brodmann 34)	0.009	
**PARIETAL**			
Left	Inferior Parietal Lobule	–	4.13(21)
**TEMPORAL**			
Left	Superior Temporal Gyrus	*0.028*	3.82(16)
Left	Parahippocampal Gyrus	*0.035*	−2.72(15); −2.70(18)
Right	Parahippocampal Gyrus	0.007	−2.72(15)
**CEREBELLAR**			
Left	Cerebellum	*0.011*	5.59(51)
Right	Cerebellum	*0.011*	4.47(41)

Regions significantly connected to the *left* STN. The 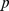-value for the structural connectivity (SC) was calculated using a 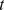-test on 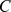, using 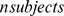 = 8. The 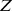-score and cluster extent (in voxels) for the functional connectivity (FC) were determined using correlations with the 10 atlas-based STN ROIs. Here only the regions with 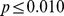 or significant functional connectivity are shown. For the latter cases, the 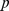-value for structural connectivity was added if lower than 0.050.

Table 2 indicates that the right STN exhibits significant connections with gray matter nuclei such as the thalamus, caudate nucleus, putamen, globus pallidus, red nucleus, and substantia nigra. Furthermore, projections to cortical areas with different functions were found, for example to the pre- and supplementary motor area (motor function), and the medial frontal and anterior cingulate cortex (limbic). With regard to the left STN, the results in Table 3 also point to connections with the gray matter nuclei and the medial frontal gyrus. No significant connections with the pre- and supplementary motor area and the anterior cingulate cortex were found. The cerebellum and temporal cortex also show significant structural connectivity to the STN.

#### Structural connectivity per voxel of the STN

**Figure 5:**
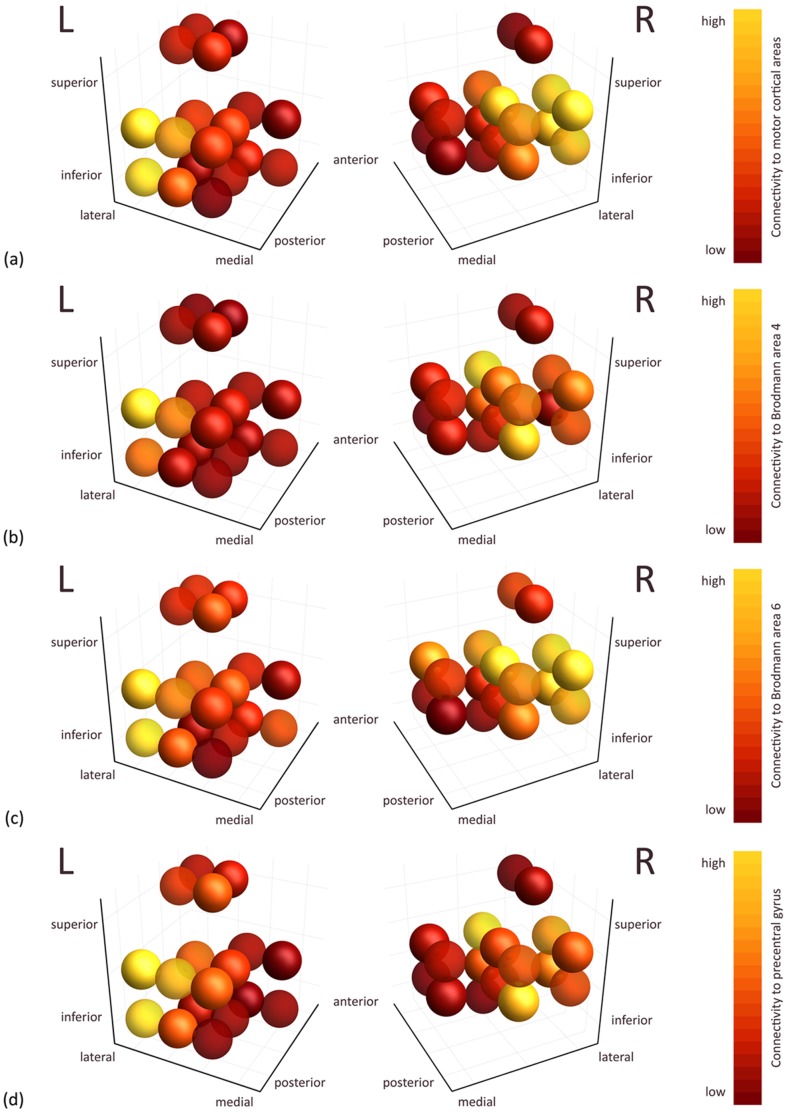
Structural connectivity to the motor cortical areas per STN voxel, cumulated over all subjects, in MNI152 atlas space. (a) Structural connectivity to the total motor cortical areas. (b) Structural connectivity to Brodmann area 4 (primary motor cortex). (c) Structural connectivity to Brodmann area 6 (pre- and supplementary motor cortex). (d) Structural connectivity to the precentral gyrus. Each sphere represents one voxel in atlas space (voxel size 2×2×2 mm) and is color-coded by the 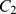 connectivity: dark red means low connectivity, while yellow means high connectivity.

The normalized maps of 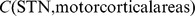, cumulated over all subjects, are visualized in Figure 5, for both the left and right STN. The images show high connectivity to the motor cortical areas in the lateral STN regions, especially for the total motor cortical areas (Figure 5(a)) and Brodmann area 6 (Figure 5(c)), while low connectivity is found medially.

### Functional Connectivity

#### Whole-brain linear regression

**Figure 6:**
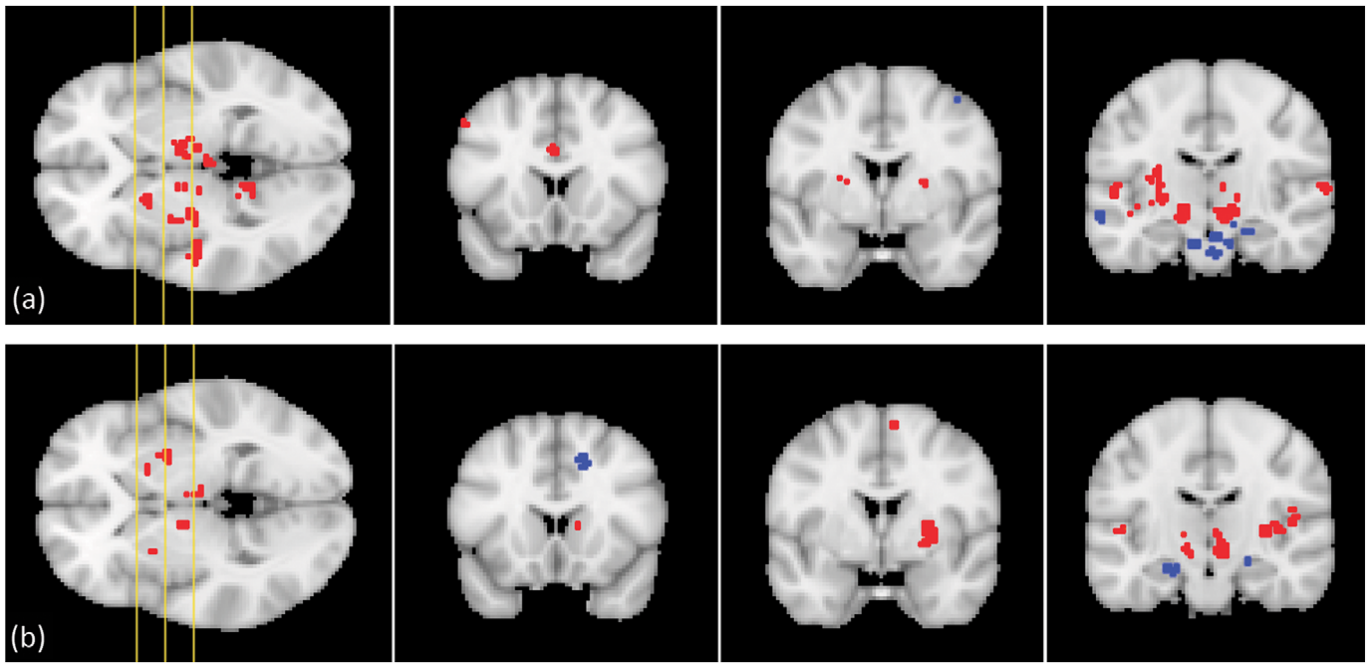
Significant functional connectivity clusters for (a) the right and (b) the left atlas-based STN ROIs, shown on three coronal slices of the MNI152 template. The yellow lines on the axial image on the left-hand side show the position of the coronal slices. Red clusters exhibit positive regression coefficients, while blue clusters yield negative coefficients.

The significant clusters were visualized onto the MNI152 template within MATLAB, as can be seen in Figure 6. The figure indicates that clusters that are significantly correlated to the STN ROIs were found in various cortical and subcortical structures. For each significant cluster, the voxel with the maximum response (maximum absolute value of test statistic) was selected. The characteristics of these voxels are specified in the fourth column of Tables 2 and 3. X, Y and Z represent the coordinates (in atlas space) of the maximum response for each cluster. The related 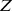-score and the cluster extent (in voxels) are given, as well as the other structures belonging to the cluster.

Tables 2 and 3 report functional connectivity of the STN ROIs with various other brain areas. The most significantly correlated structures include a group of subcortical areas such as the thalamus, caudate, putamen, and midbrain. The cerebellum is functionally connected to the STN ROIs as well. Furthermore, connected structures in the frontal cortex encompass the pre- and supplementary motor area, the medial frontal gyrus, and the cingulate gyrus, while correlations to the temporal cortex incorporate the superior temporal gyrus, the parahippocampal gyrus, and the fusiform gyrus.

#### Reverse regression per voxel of the STN

**Figure 7:**
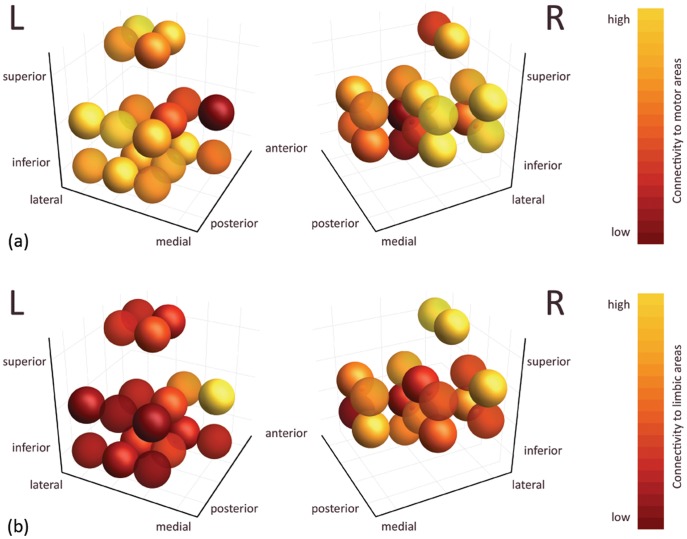
Functional connectivity per STN voxel in atlas space after applying the reverse regression procedure. (a) Connectivity to motor areas per voxel of the left and right STN, cumulated over all subjects. (b) Connectivity to limbic areas per voxel of the left and right STN, cumulated over all subjects. Each sphere in (a) and (b) represents one voxel and is color-coded by functional connectivity: dark red means low connectivity, while yellow means high connectivity.

The results of the reverse regression procedure for the right and left STN of all subjects can be seen in Figure 7. Figure 7(a) represents the functional connectivity of the STN voxels to the motor cortical areas (precentral cortex, primary motor cortex, premotor and supplementary motor area). The posterior lateral part of the STN shows the highest functional connectivity to the motor areas, while the anterior medial part yields the lowest values. Figure 7(b) shows the functional connectivity of the STN voxels to the limbic areas (amygdala, hippocampus, parahippocampal gyrus, anterior cingulate, cingulate cortex). Especially for the left STN, the posterior lateral part reveals the lowest functional connectivity to the limbic areas, while the anterior medial part returns higher values.

## Discussion

### Current Findings

In this paper, we aimed to determine the full structural and functional connectivity of the STN based on HARDI tractography and functional MRI. Streamline visualizations revealed direct (“hyperdirect” pathway) and indirect connections to the motor, cingulate, and temporal cortices. We tested the connectivity measures for significance over the group of 8 subjects. The resulting areas could be classified as belonging to a few major groups, such as the gray matter nuclei, motor cortical areas (premotor and supplementary motor area), and limbic cortex (medial frontal and cingulate cortex). Regarding the subdivision of the STN, we found high structural connectivity to the motor cortical areas in the lateral STN and low values in the medial STN parts.

The functional connectivity analysis, based on correlations in resting state BOLD signal time-series between the STN ROIs and other brain structures, supported the results described above. The resulting significant clusters for the STN ROIs again predominantly belonged to subcortical structures, the frontal cortex, temporal cortex, and cerebellum. With respect to the level of segregation, the posterior lateral part of the STN also showed the highest functional connectivity to the motor areas, while the anterior medial part yielded the lowest values. Below, we will elaborate on the correspondence of these findings with the existing literature on STN circuitry and with each other, as well as the consequent implications on the clinical practice of DBS procedures, and possible future work.

### Correspondence of Findings with Existing Literature

#### The “hyperdirect” pathway

The first evidence of the so-called “hyperdirect” pathway in the non-human primate was already provided in 1940 [64], and subsequent tracer studies extensively described the primate cortico-subthalamic projections [65]–[69]. Later, an electrophysiological study by Nambu et al. [70] confirmed the “hyperdirect” pathway. To our knowledge, our study is the first to give an indication for the existence of the “hyperdirect” pathway in humans. In 7 out of 10 STNs that exhibited a direct connection to the motor cortical areas, the “hyperdirect” pathway could be seen as a small bundle traveling along the internal capsule, a route that corresponds with the existing primate literature [14], [15]. With respect to the medial trajectory found in some volunteers, the probabilistic fiber tracking method seems to be inclined to follow anisotropic diffusion profiles from the fornix to the corpus callosum, via the dural ligaments.

#### STN connectivity with motor areas

According to tracer studies in non-human primates, within the motor circuit, the STN should exhibit connections with the following cortical areas: the primary motor cortex, premotor and supplementary motor cortex, and the somatosensory cortex [67]–[69], [71]. With respect to the deep brain, we expected to find strong connectivity with the striatum, the central and ventrolateral part of the lateral globus pallidus (GPe), the ventrolateral part of the medial globus pallidus (GPi), and the thalamus [15], [65], [72]–[74].

With respect to structural connectivity to the motor cortex, the pre- and supplementary motor area is most significant. The functional analysis yielded significant clusters for the pre- and supplementary motor area as well, but also showed connectivity to the primary motor cortex (precentral gyrus). In addition, the striatum was discovered to be strongly connected, both structurally and functionally.

#### STN connectivity with associative areas

Concerning the associative loop, we expected the STN to be connected to the orbitofrontal and dorsolateral prefrontal cortex, as well as the centromedian-parafascicular nuclei of the thalamus, the nucleus accumbens, the ventral part of the putamen and caudate nucleus, the ventral pallidum, the ventral tegmental area, and the medial part of the substantia nigra reticulata [15], [65], [67], [75]–[78].

As reported above, the gray matter nuclei present in the atlas were all found to be structurally and functionally connected to the STN. Connected associative cortical areas include the superior and middle temporal, the parahippocampal, and the fusiform gyrus. These areas often are significant for both structural and functional connectivity. In addition, the functional connectivity analysis resulted in a significant clusters in the parietal cortex.

#### STN connectivity with limbic areas

With regard to the limbic circuit, the literature reported on connections with the (para)limbic cortical areas such as the anterior cingulate and the medial orbitofrontal cortex [76]. Subcortically, the limbic loop comprises the nucleus accumbens, ventral pallidum, ventral tegmental area, substantia nigra pars reticulata, globus pallidus, thalamus, hippocampus and amygdala [76], [79].

The most significant structurally connected regions we found included the medial frontal gyrus and the cingulate cortex. Other expected limbic areas such as the substantia nigra, globus pallidus, and thalamus, were also present in the resulting tables. The cingulate and medial frontal gyri also showed significant functional connectivity. The same holds for the thalamus.

#### Segregation of motor and non-motor regions of the STN

According to review articles [11], [14], the STN is organized as follows: the medial tip of the nucleus is devoted to the limbic circuit, the associative part is situated ventrolaterally, and the motor subterritory is located at the dorsolateral side of the STN. The subdivision results based on structural connectivity to the motor cortical areas indeed show a mediolateral gradient, yielding the highest connectivity at lateral positions, where we expect the STN motor part, while connectivity in the supposed medial tip is lowest.

The findings based on our functional connectivity experiment also display this mediolateral gradient in motor and limbic connectivity. The highest connectivity to the motor regions is obtained in the posterior lateral STN part, where we expect the STN motor part, while the anterior medial part (the supposed limbic tip) contains voxels that are more connected to the limbic ROIs. The mediolateral gradient that was found indicates some level of separation between the functional parts of the STN, though no complete segregation of motor and non-motor regions was found. The latter supports the idea of open circuits, in which all pathways are partially integrated within the STN.

### Correspondence of Findings Amongst each other

When comparing the third and fourth column of Table 2 and 3, it appears there is reasonable agreement between regions that are structurally connected and regions that are functionally connected. The statistical test on the functional connectivity seems to be stricter, as it results in fewer significant regions. However, most of the regions that are functionally connected also show significant structural connectivity. Another symmetry that can be investigated is that between the right and left side of the brain. Most important structures are present in both Table 2 and Table 3, so the STN connectivity seems to be rather symmetrical in that sense. We could also evaluate the functional connectivity results with respect to positive and negative correlation coefficients. Most clusters exhibit positive correlation coefficients, however, the temporal cortex shows some negatively correlated clusters.

### Clinical Perspective

The correspondence with the existing literature on tracer and electrophysiological studies validates our structural and functional connectivity measurements. Thus, it might be feasible to assess STN connectivity in a non-invasive way. The voxel-wise connectivity assessments show that there is some separation between the different functional STN parts, and that the lateral part of the STN exhibits the highest motor connectivity. This again emphasizes that the therapeutic target for DBS is located in the dorsolateral STN part. To compensate for interindividual variations in motor connectivity, diffusion-weighted and functional MRI may assist in optimization of the patient-specific planning of the DBS procedure.

In addition, our results support the existence of the “hyperdirect” pathway, running between the motor cortical areas and the STN. The presence of the “hyperdirect” pathway in humans validates current models on cortico-basal ganglia circuits. This pathway could be used in electrophysiological studies to target the STN motor part during DBS procedures.

### Future Work

The pipeline that we used for the calculation of structural and functional connectivity can still be improved in multiple ways. First of all, for the acquisition should be looked into. On the one hand, a higher spatial resolution would probably improve the separation of the STN motor and non-motor parts. On the other hand, the MRI acquisition should be clinically feasible, so we should assess the number of gradient directions and time points absolutely necessary. If we would extend the acquired data with an isotropic structural (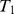-weighted) image of the brain, this could enhance the image registration (in comparison to the currently used data with limited field-of-view and thick slices).

Second, the atlas-based STN segmentation is sensitive to registration inaccuracies. In addition, we could question the inherent precision of this ROI for the left STN, as it contains two parts that only touch each other at a corner point of two voxels, instead of sharing a voxel edge or face. The use of the atlas-based STN could be avoided by using 7 T MRI for the localization of the STN [80]–[82]. The evidence for the “hyperdirect” pathway is also susceptible to registration inaccuracies, via the atlas segmentations of gray matter nuclei that should be bypassed by this pathway.

Furthermore, validation of diffusion-weighted data in itself is a well-known issue. We can only compare the found projections with the results of electrophysiological studies in humans, or tract tracing experiments in animals, but a ground truth for all white matter tracts (also smaller bundles) in humans is not readily available. Such a ground truth could be used to correct the structural connectivity results, based on a probabilistic fiber tracking method that inherently finds all possible pathways, for non-existing anatomical connections. We did not perform such a correction in this study and for instance found some contralateral structural connections of the STN. However, according to literature on STN circuitry [15], [71], no direct contralateral cortico-subthalamic pathways are to be expected.

Future work could include joining all available information per voxel of the STN (structural connectivity, functional connectivity, local diffusion information) in order to obtain a more robust conclusion on the level of segregation of the motor, limbic, and associative regions of the STN. To this end, the connectivity of each STN voxel to the different functional parts of the globus pallidus and striatum could also be taken into account [39]. Another way to achieve more robust results would involve the inclusion of more subjects. Ultimately, similar work remains to be done on PD patients to be able to validate the direct and reverse regression procedure for DBS planning.

### Conclusions

Through analysis of the structural and functional connectivity of the STN based on HARDI and resting state fMRI data, we were able to confirm the STN’s connections to motor, associative, and limbic areas that have been found before by means of neuronal tract tracing and electrophysiological studies. Furthermore, we produced evidence for the existence of the “hyperdirect” pathway from motor cortex to STN in humans. We also reported that the connectivity of distinct STN voxels to the motor cortical areas increased when going from the medial to the lateral STN, though a clear segregation was not seen. This gradient in connectivity might indicate that the STN motor part and therefore the therapeutic target for STN DBS is located dorsolaterally.

While improvements could be made on the amount of data and the registration and validation steps, this work is a promising step towards the use of diffusion-weighted and functional MRI for the segmentation of the STN motor part, which in turn could optimize patient-specific STN DBS planning.
